# Supplementary material for: Seminoma and Embryonal Carcinoma Footprints Identified by Analysis of Integrated Genome-Wide Epigenetic and Expression Profiles of Germ Cell Cancer Cell Lines
Source: PLoS One. 2014 Jun 2;9(6):e98330. doi: 10.1371/journal.pone.0098330 (PMC4041891; doi:10.1371/journal.pone.0098330)
Supplement: File S1 — ZIP file containing DMRforPairs output for significant regions. Please start from the html files. (ZIP) [file pone.0098330.s008.zip › figures/10794.pdf]

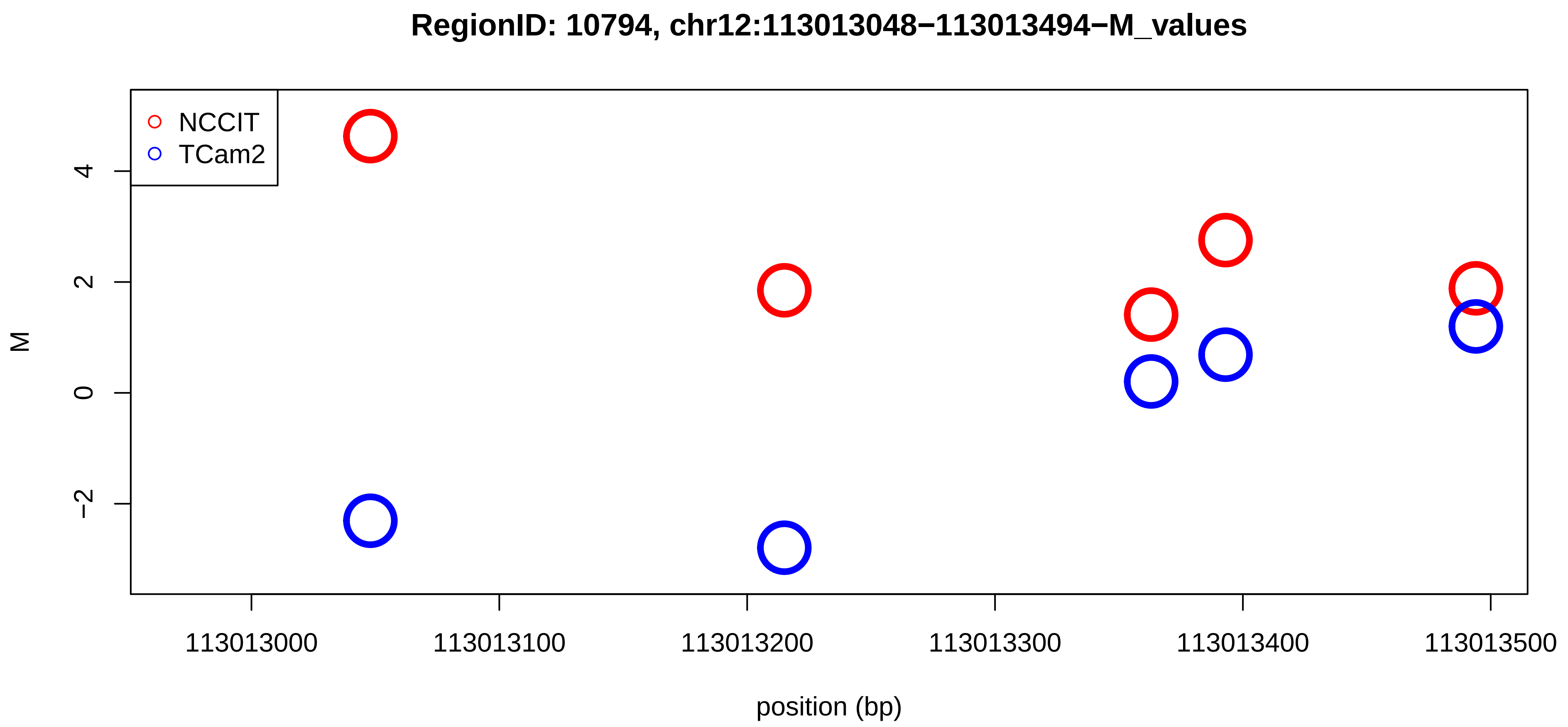

RegionID: 10794, chr12:113013048−113013494−M_values
NCCIT
TCam2
M
−2
0
2
4
113013000
113013100
113013200
113013300
113013400
113013500
position (bp)

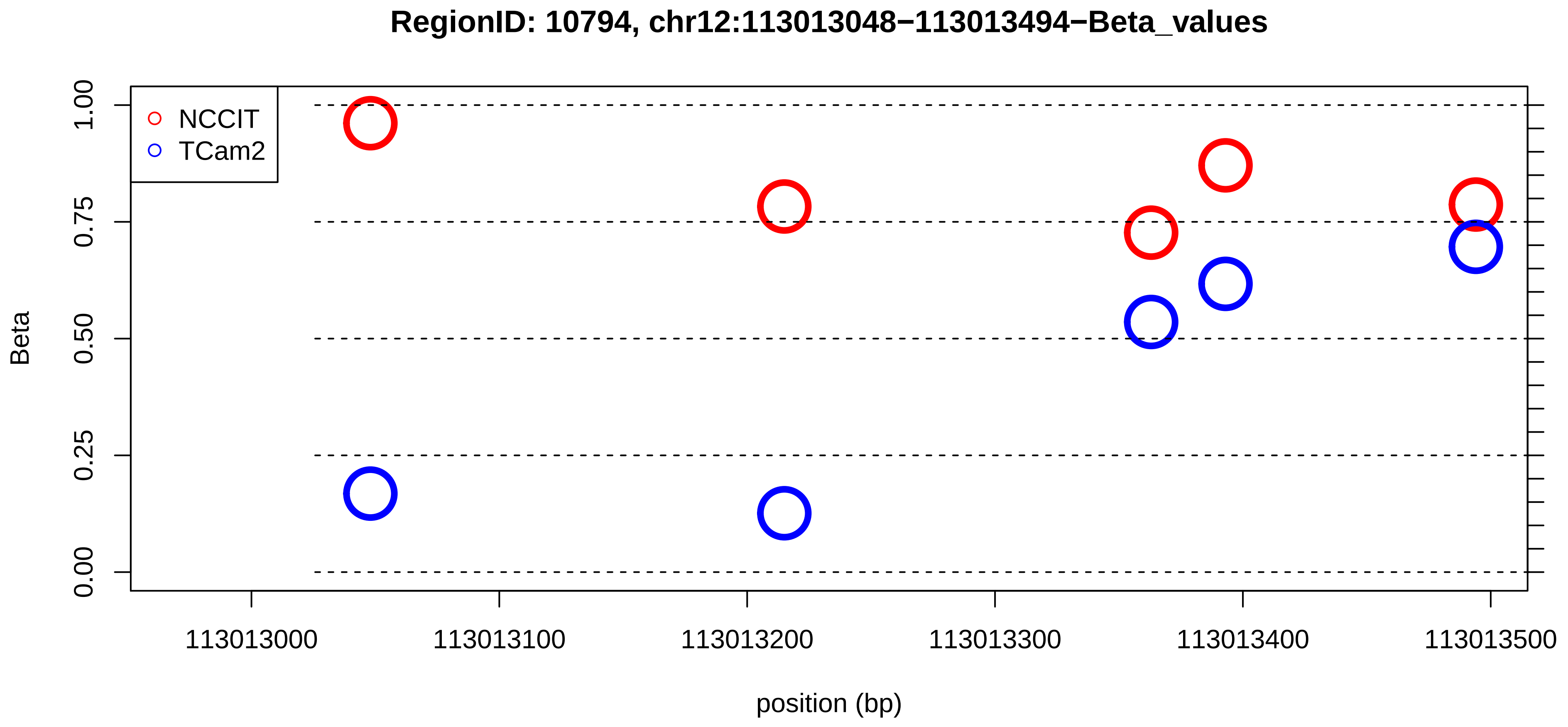

RegionID: 10794, chr12:113013048−113013494−Beta_values
NCCIT
TCam2
Beta
0.00
0.25
0.50
0.75
1.00
113013000
113013100
113013200
113013300
113013400
113013500
position (bp)
